# Genetic and phenotypic assessment of the antimicrobial activity of three potential probiotic lactobacilli against human enteropathogenic bacteria

**DOI:** 10.3389/fcimb.2023.1127256

**Published:** 2023-02-08

**Authors:** Despoina Eugenia Kiousi, Christos Efstathiou, Vasilis Tzampazlis, Stavros Plessas, Maria Panopoulou, Maria Koffa, Alex Galanis

**Affiliations:** ^1^ Department of Molecular Biology and Genetics, Faculty of Health Sciences, Democritus University of Thrace, Alexandroupolis, Greece; ^2^ Department of Agricultural Development, Democritus University of Thrace, Orestiada, Greece; ^3^ Department of Medicine, Faculty of Health Sciences, Democritus University of Thrace, Alexandroupolis, Greece

**Keywords:** lactobacilli, probiotics, antimicrobial, competitive exclusion, biofilms, enteropathogens, confocal microscopy, *in silico* analysis

## Abstract

**Introduction:**

Lactobacilli are avid producers of antimicrobial compounds responsible for their adaptation and survival in microbe-rich matrices. The bactericidal or bacteriostatic ability of lactic acid bacteria (LAB) can be exploited for the identification of novel antimicrobial compounds to be incorporated in functional foodstuffs or pharmaceutical supplements. In this study, the antimicrobial and antibiofilm properties of *Lactiplantibacillus pentosus* L33, *Lactiplantibacillus plantarum* L125 and *Lacticaseibacillus paracasei* SP5, previously isolated form fermented products, were examined, against clinical isolates of *Staphylococcus aureus*, *Salmonella enterica* subsp. *enterica* serovar Enteritidis and *Escherichia coli*.

**Methods:**

The ability of viable cells to inhibit pathogen colonization on HT-29 cell monolayers, as well as their co-aggregation capacity, were examined utilizing the competitive exclusion assay. The antimicrobial activity of cell-free culture supernatants (CFCS) was determined against planktonic cells and biofilms, using microbiological assays, confocal microscopy, and gene expression analysis of biofilm formation-related genes. Furthermore, *in vitro* analysis was supplemented with *in silico* prediction of bacteriocin clusters and of other loci involved in antimicrobial activity.

**Results:**

The three lactobacilli were able to limit the viability of planktonic cells of *S. aureus* and *E. coli* in suspension. Greater inhibition of biofilm formation was recorded after co-incubation of *S. enterica* with the CFCS of *Lc. paracasei* SP5. Predictions based on sequence revealed the ability of strains to produce single or two-peptide Class II bacteriocins, presenting sequence and structural conservation with functional bacteriocins.

**Discussion:**

The efficiency of the potentially probiotic bacteria to elicit antimicrobial effects presented a strain- and pathogen-specific pattern. Future studies, utilizing multi-omic approaches, will focus on the structural and functional characterization of molecules involved in the recorded phenotypes.

## Introduction

Pathogen resistance to common antibiotics is a serious health threat, causing significant morbidity and mortality worldwide (Wang et al., 2020). The excessive use of antibiotics and the lack of stewardship have resulted in the emergence and spread of resistant and multi-resistant pathogens to the nosocomial environment and community (Bonnet et al., 2019). Furthermore, pathogens capable of forming biofilms, three dimensional microbial structures with heightened resistance to antibiotics, pose an additional threat to immunocompromised and frail individuals (Crabbé et al., 2019). The need to identify novel antibiotic and antibiofilm agents has steered research towards the characterization of the antimicrobial properties of non-pathogenic bacteria and fungi (Wiese and Imhoff, 2019). Microorganisms resort to the production of antimicrobial compounds for the survival of their kin, when competing for finite resources in complex microbial communities (Mullis et al., 2019). These compounds can either be proteins or secondary metabolites, including ethanol, acidic compounds, and reactive oxygen species (ROS) exerting bacteriostatic or bactericidal effects (Fuochi et al., 2019). Furthermore, some bacteria can also act by hijacking quorum sensing signals and signaling pathways involved in biofilm formation and maturation (Algburi et al., 2017).

Members of the emended *Lactobacillus* genus have long been investigated for their ability to limit the proliferation of distantly or closely related bacteria (Arqués et al., 2015). Some lactobacilli are defined as probiotics; non-pathogenic microbes that could be advantageous, when consumed in adequate amounts (Hill et al., 2014). The antimicrobial activity of probiotics is mainly mediated by the production of lactic acid, and thus the acidification of the matrix, however some strains may additionally code for broad or narrow spectrum bacteriocins (Iseppi et al., 2019; Guo et al., 2020). Furthermore, competition for nutrients and adhesion sites on host mucosa, or induction of proinflammatory responses for pathogen clearance have also been observed, *in vitro* and *in vivo* (Walsham et al., 2016; Tuo et al., 2018). Therefore, the inclusion of these strains in fermented foods can provide additional protection against contamination with foodborne (Ayala et al., 2019) or other clinically relevant pathogens (Lee et al., 2020; Stašková et al., 2021). Today, the incorporation of multi-omic approaches in the microbiology field has streamlined the identification of novel probiotic strains and the characterization of their antimicrobial capacity at the molecular and cellular level (Kiousi et al., 2021). Indeed, whole genome sequencing (WGS) technologies and robust predictive algorithms can be utilized to mine loci of interest, providing new leads for further preclinical and clinical validation (Bindu and Lakshmidevi, 2021).

In the present study, the antimicrobial capacity of three wild type, potentially probiotic LAB strains, *Lactiplantibacillus pentosus* L33, *Lactiplantibacillus plantarum* L125 and *Lacticaseibacillus paracasei* SP5, was examined, through a series of interconnected approaches. Of note, WGS, comparative genomic analysis and annotation of genes conferring the putative probiotic phenotype of *Lp. pentosus* L33 (Stergiou et al., 2021), *Lp. plantarum* L125 (Tegopoulos et al., 2021) and *Lc. paracasei* SP5 (Kiousi et al., 2022) have been previously published. Thus, here, we sought to expand the bioinformatic analysis and validate the antimicrobial potential of the strains, against clinical isolates of *Staphylococcus aureus, Salmonella enterica* ssp *enterica* serovar Enteritidis and *Escherichia coli.* The selected pathogens are responsible for intestinal or extraintestinal infections, while their ability to form biofilms contributes to the development of persistent infections, that do not respond to antibiotic therapy (Fleckenstein et al., 2021). To this aim, competition between lactobacilli and pathogens for nutrients and attachment sites, and the inhibitory effects of whole cells and of the CFCS, on the viability of planktonic cells and biofilm formation was investigated, using microbiological assays and confocal microscopy. The effects of CFCS on the expression levels of biofilm formation-related genes were studied at the transcriptome level. Finally, the genetic clusters identified in the genome of the three strains were examined for their completeness, with the employment of comparative genomics methods, and the physicochemical properties of the core peptides were predicted, using established algorithms.

## Materials and methods

### Bacterial strains and culture conditions

*Lc. paracasei* SP5 was previously isolated from kefir grains (Mantzourani et al., 2019), whereas *Lp. pentosus* L33 and *Lp. plantarum* L125 were isolated from traditional fermented meat products (Pavli et al., 2016) and provided by the Institute of Technology of Agricultural Products, Hellenic Agricultural Organization DIMITRA, Likovrisi, Attiki, Greece. All lactobacilli were cultivated O/N in de Man, Rogosa and Sharp (MRS) broth at 37°C, under anaerobic conditions for 20 h. Clinical isolates of *Staphylococcus aureus*, *Salmonella enterica* ssp. *enterica* serovar Enteritidis and *Escherichia coli* were isolated from clinical specimens of stools, at the Laboratory of Clinical Microbiology, University Hospital of Alexandroupolis. Bacterial identification was based on conventional methods, the automated system Vitek II (Biomerieux, France) and the VITEK^®^ MS automated mass spectrometry microbial identification system (Biomerieux, France). Pathogens were maintained in Tryptic Soy Broth (TSB, Condalab, Madrid, Spain) at 37°C under anaerobic conditions. Tryptone soya agar (TSA) (Condalab, Madrid, Spain) and McConkey agar (VWR, Radnor, PA, USA) were used to culture *S. aureus* or *S. enterica* and *E. coli*, respectively, for colony enumeration.

### Human colorectal adenocarcinoma cell line

The human colorectal adenocarcinoma cell line HT-29 was purchased from ATCC. Cells were maintained in Roswell Park Memorial Institute GlutaMAX™ (RPMI)-1640 medium, containing 10% fetal bovine serum (FBS), 100 μg/mL streptomycin and 100 U/mL penicillin (all from Thermo Fisher Scientific, Waltham, MA, USA). For HT-29-bacteria co-incubations, a modified RPMI-1640 medium consisted of 10% FBS and 20mM 4-(2-hydroxyethyl)-1- piperazineethanesulfonic acid (HEPES; all from Thermo Fisher Scientific) was used. Cells were incubated in a humidified atmosphere at 37°C, 5% CO_2_ under sterile conditions.

### Well diffusion assay

The antimicrobial activity of the viable potentially probiotic cultures against the enteropathogens was investigated using the well diffusion assay (Balouiri et al., 2016). More specifically, TSA plates were inoculated with fresh O/N cultures of the pathogenic bacteria, using a sterile swab. Then, wells were punctured into the agar (0.7 cm) and filled with 100 μL of lactobacilli (approximately 10^9^ CFU/mL). Plates were left at RT for 1 h to facilitate diffusion and were, subsequently, incubated at 37°C for 20 h. Inhibition zones were measured the next day.

### Auto- and co-aggregation capacity

Fresh O/N cultures of lactobacilli and pathogens were utilized to investigate the auto-aggregation and co-aggregation capacity of strains, following a previously published protocol (Prabhurajeshwar and Chandrakanth, 2017), with minor modifications. To test for co-aggregation capacity, 10^8^ CFU/mL of lactobacilli were mixed with 10^8^ CFU/mL of pathogens. Lactobacilli (10^8^ CFU/mL) and pathogens (10^8^ CFU/mL) were separately tested for auto-aggregation. Samples were incubated for 4 h at RT under strictly static conditions. The upper layer (500 μL) was collected at 0 and 4 h of incubation to be subjected to spectrophotometry at 620 nm. Results are expressed as: Co-aggregation (%) = [(A_1_ + A_2_)/2 – A_mix_(A_1_ + A_2_)/2] × 100, where A_1_ and A_2_ represent the absorbance at 620 nm of pathogen monocultures and lactobacilli (auto-aggregation), respectively, and A_mix_ the absorbance of the mixed culture (co-aggregation). Auto-aggregation was calculated using the formula: 1- (A_t=0h_/A_t=4h_) × 100, where A_t=0h_ and A_t=4h_ refer to absorbance at 620 nm of the monocultures at 0 and 4 h, respectively.

### Attachment competition assay

The ability of the potentially probiotic strains to inhibit pathogen adhesion on HT-29 cell monolayers was determined after 4 h co-incubations. Briefly, HT-29 cells were seeded in 24-well plates at a density of 4 ×10^5^ cells per well and were incubated until the formation of a monolayer (100% confluency), following a previously published protocol (Plessas et al., 2020). Then, cells were treated with lactobacilli and/or pathogens at a concentration of 10^8^ CFU/mL for 4 h. Control samples were incubated with pathogens alone for 4 h, under the same conditions. The monolayers were washed twice with Phosphate-Buffered Saline (PBS) (Thermo Fischer Scientific) to remove unattached bacteria and cells were detached using 1% v/v Trypsin (Thermo Fischer Scientific). The suspension was serially diluted in 1× Ringer’s solution (Lab M, Lancashire, United Kingdom) and plated onto agar plates: TSA plates for *S. aureus* and McConkey agar plates for *S. enterica* and *E. coli* enumeration. Plates were incubated at 37°C, under anaerobic conditions, until the formation of visible colonies. Attached bacteria on epithelial cells are expressed as Log CFU/mL.

### *In vitro* antimicrobial activity of viable lactobacilli and CFCS against planktonic cells

The antimicrobial potential of viable lactobacilli was determined using a previously published protocol with minor modifications (Ayala et al., 2019). Briefly, pathogens (10^8^ CFU/mL) and lactobacilli (10^8^ CFU/mL) were co-incubated for 24 h, at 37°C under anaerobic conditions in TSB (Condalab). The next day, the bacterial suspension was serially diluted in 1× Ringer’s solution (Lab M) and spread in agar plates, for colony enumeration. The investigation of the antimicrobial activity of secreted metabolites was performed as described previously, with minor modifications (Forestier et al., 2001). Lactobacilli were grown for 22 h in MRS and CFCS (10 mL) was collected by centrifugation at 4,000 × g for 10 min and filtered through an 0.2 μm filter. Native CFCS (pH 4.2) or CFCS adjusted to pH 6.2 with NAOH 2M, were used to determine the contribution of pH to the antimicrobial activity of the tested strains. Similarly, native (RT) or heat-treated CFCS (incubation at 100°C for 30 min), were used to determine the stability of the bioactive antimicrobial compounds at denaturing temperature. To this aim, 10^8^ CFU/mL of pathogens were added to the CFCS and were left to incubate for 24 h in sterile tubes. MRS adjusted at the appropriate pH or heat-treated MRS at 100°C were used as a negative control. After the end of the incubation period, samples were vortexed and transferred to a 96-well plate for absorbance reading at 620 nm, using a microplate reader (EnSpire Multimode Plate Reader, PerkinElmer, Waltham, MA, United States). Results are expressed as the percentage (%) = [(Sample OD_620_-Media blank OD_620_)/(Mean control OD_620_-Media blank OD_620_)] × 100.

### Antibiofilm activity of CFCS

The ability of CFCS to limit biofilm formation was investigated using a microbiological assay, as described previously, with minor modifications (Sabbatini et al., 2020). Briefly, 10^8^ CFU/mL of fresh O/N cultures of pathogens (100 μL) were co-incubated with 100 μL adjusted CFCS (pH 6.2) in a 96-well plate for 24 h. For the estimation of viable cells, biofilms were washed twice post-treatment with PBS, and were mechanically disrupted. The suspension was serially diluted in 1× Ringer’s solution (Lab M) and spread on agar plates for colony enumeration. Plates were incubated at 37°C under anaerobic conditions until the formation of visible colonies. Viable bacteria are presented as Log CFU/mL.

### Confocal microscopy

Confocal microscopy was used for the visualization of the inhibitory effect of CFCS on biofilm formation. To this end, pathogens (10^8^ CFU/mL) were seeded on No. 1.5 coverslips and were treated with either MRS (control) or CFCS (50% v/v) adjusted to pH 6.2 for 24 h. Then, biofilms were washed twice with PBS and incubated with 10 μM carboxyfluorescein succinimidyl ester (CFSE) stain (BD Biosciences, Franklin Lakes, NJ, USA) and 1 μg/mL Hoechst 33342 dye (Biotium, San Francisco, CA, USA) for 1 h. Coverslips were washed three times with PBS and fixed in 4% paraformaldehyde (PFA) (AppliChem, Darmstadt, Germany) in PHEM solution [25 mM HEPES, 10 mM EGTA (Merck Millipore, Burlington, MA, USA), 60 mM PIPES, 2 mM MgCl2 (Applichem) pH 6.9], for 12 min at RT, followed by three washes with PBS. Finally, coverslips were mounted in mowiol 4-88 (AppliChem) medium.

Image acquisition was performed on a customized Andor Revolution Spinning Disk Confocal system (Yokogawa CSUX1; Yokogawa, Tokyo, Japan), built around an Olympus IX81 (Olympus Shinjuku, Tokyo, Japan), with 60x 1.42NA oil lens (UPlanXApo; Olympus Shinjuku, Tokyo, Japan) and a digital camera (Andor Zyla 4.2 sCMOS; Andor Technology Ltd., Belfast, Northern Ireland). The system was controlled by Andor IQ3.6 software (Andor Technology). Images were acquired as z-stacks with a z-step of 0.5 μm, through the entire volume of the biofilm. For each image, the maximum projection of z-stacks was generated, and the background was subtracted using a custom script in ImageJ (National Institute of Health, United States).

### RNA extraction, cDNA synthesis and gene expression analysis with RT-qPCR

Gene expression analysis at the transcriptome level was employed to examine changes in the production of biofilm-formation-related genes after exposure to CFCS. To this aim, 10^8^ CFU/mL of fresh O/N cultures of the pathogenic bacteria were seeded in a 100 mm well. Adjusted CFCS (50% v/v, pH 6.2) was added simultaneously, and the cells were left to incubate for 24 h. The next day, the bacterial suspension was discarded, and the adhered bacteria were washed with PBS. Then, biofilms were scraped off, and the resulting suspension was centrifuged at 8,000 × g for 5 min. *S. aureus* cells were disrupted using a lysis buffer containing 1 M Tris-Cl (pH 8), 0.5 M EDTA (pH 8), 10% v/v Triton-X100 and 100 mg/mL lysozyme. *S. enterica* and *E. coli* were resuspended at the same lysis buffer, without the addition of lysozyme, and were incubated at 37°C for 30 min. Subsequently, cells were lysed by sonication. Finally, 1 mL of ice-cold Trizol (Sigma-Aldrich, Saint Louis, MO, USA) was added to each sample. RNA extraction was performed according to the manufacturer’s instructions. RNA integrity was investigated with agarose gel electrophoresis. 100 ng of whole RNA was used as a substrate for cDNA synthesis (Invitrogen, Waltham, MA, USA). Gene expression analysis was performed using Real-time PCR. Briefly, reactions were performed on a StepOne PCR System in MicroAmp^®^ Fast Optical 48-Well Reaction Plates (both from Thermo Fisher Scientific), using the KAPA SYBR^®^ FAST qPCR Kit (Kapa Biosystems, Wilmington, MA, USA), following manufacturer’s instructions. Each reaction mixture (20 μL) consisted of 10 μL SYBR Premix, 0.4 μL of forward and reverse primer, 2 μL cDNA and 7.2 μL ddH_2_O. The PCR conditions were: 95°C for 3 min followed by 40 cycles of 95°C for 15 sec and 60°C for 1 min. All reactions were performed in duplicates and each experiment included two non-template, negative controls for each primer used. For the relative quantification of gene expression, the formula RQ=2^-ΔΔCt^ was used. Primer sequences are shown in **Table 1**.

**Table 1:** Primers used for quantitative real-time PCR analysis.

Gene	Forward Primer Sequence	Reverse Primer Sequence	Protein Function
*icaD*	5’-ACCCAACGCTAAAATCATCG-3’	5’-ACCCAACGCTAAAATCATCG-3’	Biofilm formation
*icaA*	5’-CGCAGCAGTAGTTCTTGTCG-3’	5’-GGTATTCCCTCTGTCTGGGC-3’	Biofilm formation
*eno*	5’-AAACTGCCGTAGGTGACGAA-3’	5’-TGTTTCAACAGCATCTTCAGTACCTT-3’	Matrix attachment
*fnbpA*	5’-CGCGGATCCGGTACAGATGTAACAAGTAAAG-3’	5’-GACGCGTCGACTTAATTCGGACCATTTTTCTCATT-3’	Fibronectin-binding protein
gyrB*	5’-TTATGGTGCTGGGCAAATACA-3’	5’-CACCATGTAAACCACCAGATA-3’	Housekeeping gene (DNA replication)
*csgA*	5’-TTACTGTCGGCCAATACGGC-3’	5’-CAAAACCAACCTGACGCACC-3’	Curli fimbriae production
*csgD*	5’-CCACGTGTTCCTGGTCTTCA-3’	5’-CGGCCGGTTGCATTGTTTTA-3’	Biofilm formation regulator
*cxpR*	5’-CATCAGGGCTATTTTGCGCC-3’	5’-AGGCTTAGCGCATCGACTTC-3’	Biofilm formation regulator
*bscQ*	5’-GCGAGTTTGTGGCGATCTTC-3’	5’-TCAGGAACCAGCCCATTGTC-3’	Cellulose synthase
*csrA*	5’-CGGGATACAGAGAGACCCGA-3’	5’-GAGGGTCTCACCAACTCGAC-3’	Negative regulator of biofilm formation
*luxS*	5’-ACGCCATTACCGTTAAGATG-3’	5’- AGTGATGCCAGAAAGAGGGA-3’	Quorum sensing
*pgaA*	5’- AGGCTTATGTTCGCTGGTATC-3’	5’- TAGTATGGAGTGTCGTGTTCTG-3’	PGA biosynthesis & transport
*cpxA*	5’-TCTGGATAGCGAACAGCGTC-3’	5’-TAAATCGTTGGGCGGATCGT-3’	Adhesion on hydrophobic surfaces
gyrB**	5’-CTGTTCCTGCTTACCTTTCTTCAC-3’	5’-ACGCGTCTGTTGACCTTCTTC-3’	Housekeeping gene (DNA replication)

PGA, Poly-beta-1,6-N-acetyl-D-glucosamine. *Primer sequence used for *S. aureus*; **Primer sequence used for *S. enterica* and *E. coli*.

### *In silico* analysis

To investigate the genetic basis of the antimicrobial potential of the strains of interest, a comprehensive bioinformatic analysis with established predictive algorithms was performed. Putative bacteriocin clusters were predicted using BAGEL4 (van Heel et al., 2018), BLASTp and local BLASTp+ (Camacho et al., 2009). More specifically, these algorithms were used to identify homologous genes to putative bacteriocin peptides, immunity proteins, transporters, and pheromone peptides. Additionally, the database Bactibase was utilized to mine sequences of functionally characterized bacteriocins (Hammami et al., 2010). Amino acid sequences of closely related species were aligned using CLUSTALW (Thompson et al., 1994) and T-COFFEE (Notredame et al., 2000) and phylogenetic trees were built using MEGA-X (version 11) (Tamura et al., 2021). More specifically, the aligned sequences were used as input to construct a tree with the Neighbor-Joining method and bootstrap replication values of 1000. The publicly available iTol server (Letunic and Bork, 2016) was used for the visualization of the resulting phylogenetic tree. The prediction of the topology and physicochemical properties of proteins was performed using DeepTHMM (Hallgren et al., 2022) and ProtParam (Gasteiger et al., 2005), respectively. InterPro (Blum et al., 2021) and Signal 6.0 (Teufel et al., 2022) were employed to investigate the family of the identified proteins, related motifs, and N-terminal signals. Structure predictions were performed using CollabFold, the publicly available version of AlphaFold2 (Mirdita et al., 2022) and Chimera version 1.16 was employed for structure superimpositions (Pettersen et al., 2004). Finally, assignment into Kyoto Encyclopedia of Genes and Genomes (KEGG) orthologous (KO) groups (Kanehisa et al., 2016) was performed to annotate pathways involved in the antimicrobial phenotype.

### Statistical analysis

For the statistical analysis of the experimental data, Student’s T-test was performed using GraphPad PRISM 9 (GraphPad Software Inc., CA, USA). All experiments were performed in triplicate unless otherwise stated. Results are represented as mean ± standard deviation. A *p*-value < 0.05 was considered statistically significant.

### Availability of data

The WGS of *Lc. paracasei* SP5, *Lp. pentosus* L33, *Lp. plantarum* L125 is available at the DDBJ/ENA/GenBank under the accession numbers: JAHKRU000000000.1, JAIGOE000000000.1 and JAKJPP000000000.1, respectively.

### Ethics statement

This study was approved (24-10/12/2022) by the Institution Review Board of the General University Hospital of Alexandroupolis, Greece.

## Results

### Antimicrobial activity of viable lactobacilli against planktonic pathogenic bacteria

**Figure 1:**
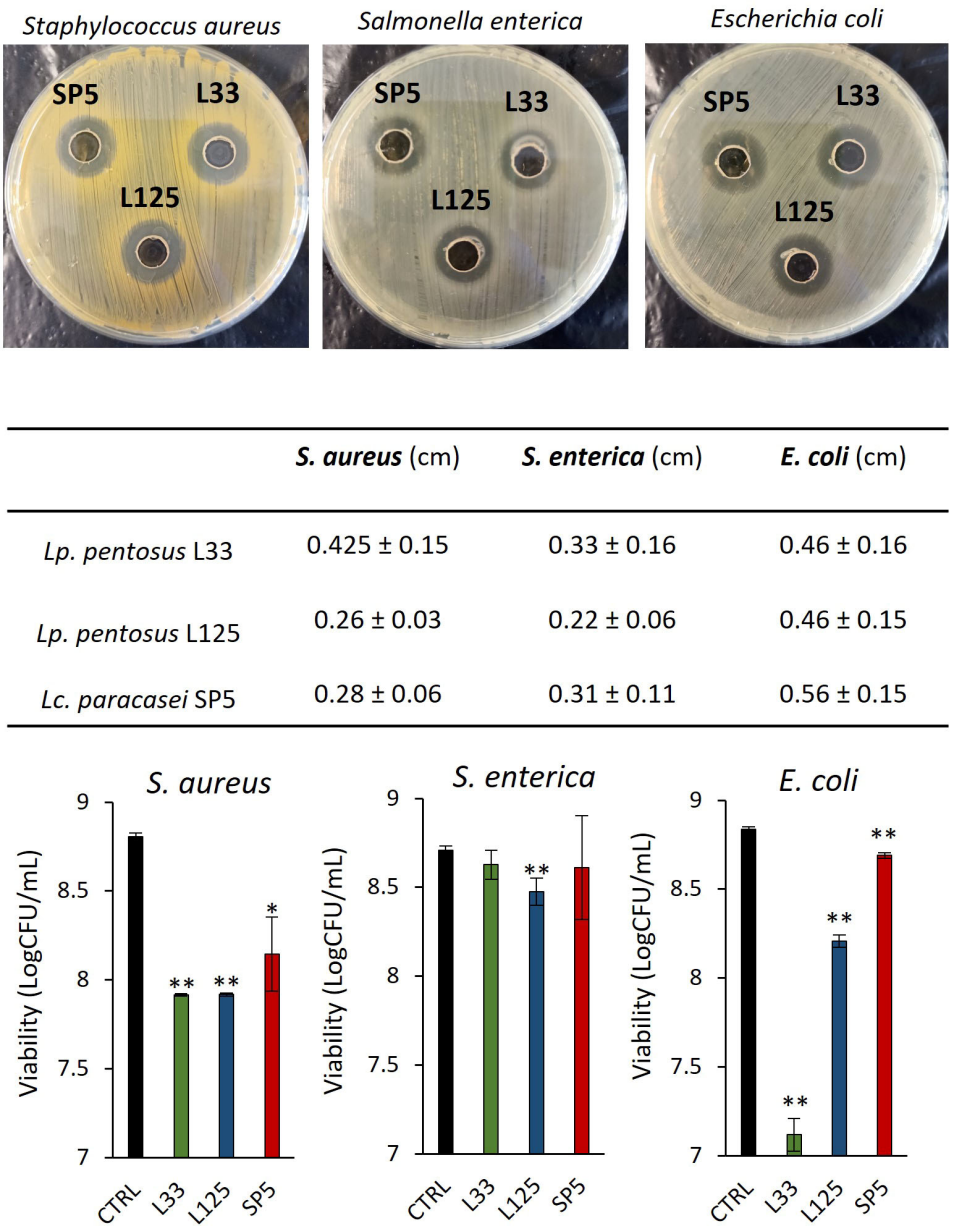
Evaluation of the antimicrobial activity of viable potential probiotic LAB strains against *S. aureus, S. enterica* and *E*. *coli.* **(A)** Representative photos of inhibition zones of fresh O/N cultures of *Lp. plantarum* L125, *Lp. pentosus* L33 and *Lc. paracasei* SP5 against *S. aureus*, *S. enterica* and *E*. *coli*, using the agar well diffusion assay. **(B)** Zone of inhibition in cm. Results are expressed as the mean ± standard deviation of three independent experiments. **(C)** Antimicrobial activity of viable lactobacilli against planktonic pathogens in co-culture for 24 h. Results are expressed as mean ± standard deviation of three independent experiments. *p < 0.05, **p < 0.01, compared to control, untreated samples.

A preliminary examination of the antimicrobial potential of *Lp. pentosus* L33, *Lp. plantarum* L125 and *Lc. paracasei* SP5, against clinical isolates of *S. aureus, S. enterica* ser. Enteritidis and *E. coli*, was performed, using the agar well diffusion assay. It was shown that the three strains inhibited the growth of the pathogens with variable efficiency (**Figures 1A, B**). The most profound effect was induced by *Lc. paracasei* SP5 against *E. coli* in the well diffusion assay, while in suspension *Lc. paracasei* SP5 was most effective against *S. aureus* (**Figure 1C**). In this context, all putative probiotic strains exerted antimicrobial effects after 24 h co-incubation with *S. aureus* or *E. coli* (*p* < 0.05), in suspension. The highest inhibitory activity against *S. aureus* was induced by co-incubations with *Lp. pentosus* L33 or *Lp. plantarum* L125 (~1.5 log reduction), while co-incubation of *E. coli* with *Lp. pentosus* L33, limited pathogen viability by ~1.7 log. Of note, *S. enterica* was resistant to *Lp. pentosus* L33 and *Lc. paracasei* SP5 co-incubation, whereas a significant reduction was observed with *Lp. plantarum* L125.

### Competition for adherence and co-aggregation ability of lactobacilli

**Figure 2:**
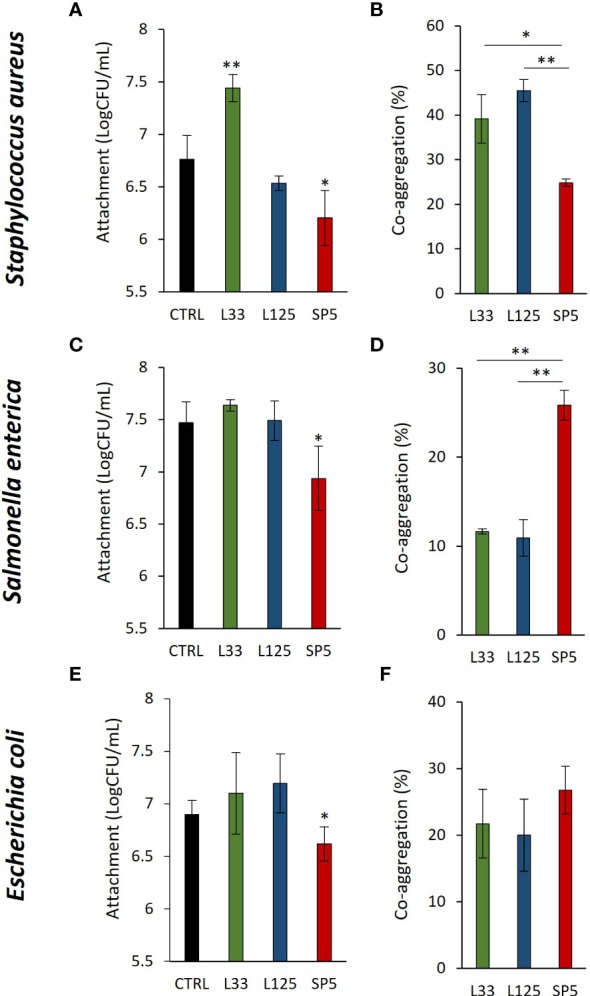
Competitive exclusion and co-aggregation capacity of the lactobacilli with the pathogens. **(A, C, E)** Competition between the live lactobacilli and **(A)** *S. aureus*, **(C)** *S. enterica* or **(E)** *E. coli* for attachment on the human colon adenocarcinoma cell line, HT-29, after 4 h of co-incubation. Results are expressed as the mean ± standard deviation of three independent experiments. *p < 0.05 compared to untreated control. **(B, D, F)** Investigation of the co-aggregation capacity of lactobacilli with pathogens after 4 h co-incubation in suspension. Results are expressed as the mean ± standard deviation of three independent experiments. *p < 0.05, **p < 0.01.

Next, we determined lactobacilli-pathogen competition for adherence on HT-29 cell monolayers. It was found that *Lc. paracasei* SP5 significantly limited adherence of all pathogens on the eukaryotic monolayers (*p* < 0.05, **Figures 2A, C, E**). On the contrary, *Lp. pentosus* L33 promoted the adhesion of *S. aureus* on HT-29 cells (**Figure 2A**). Next, we sought to determine the co-aggregation capacity of lactobacilli with the three pathogens. As shown in **Figures 2B, D, F**, *Lc. paracasei* SP5 demonstrated similar co-aggregation efficiency with all pathogenic bacteria, while *Lp. pentosus* L33 and *Lp. plantarum* L125 showed higher preference for the gram-positive *S. aureus* (**Figure 2B**)*. Lp. plantarum* L125 exhibited the highest auto-aggregation capacity among the lactobacilli. The auto-aggregation capacity of the three lactobacilli and pathogens is presented in **Supplementary Figure 1**.

### Antimicrobial capacity of CFCS

**Figure 3:**
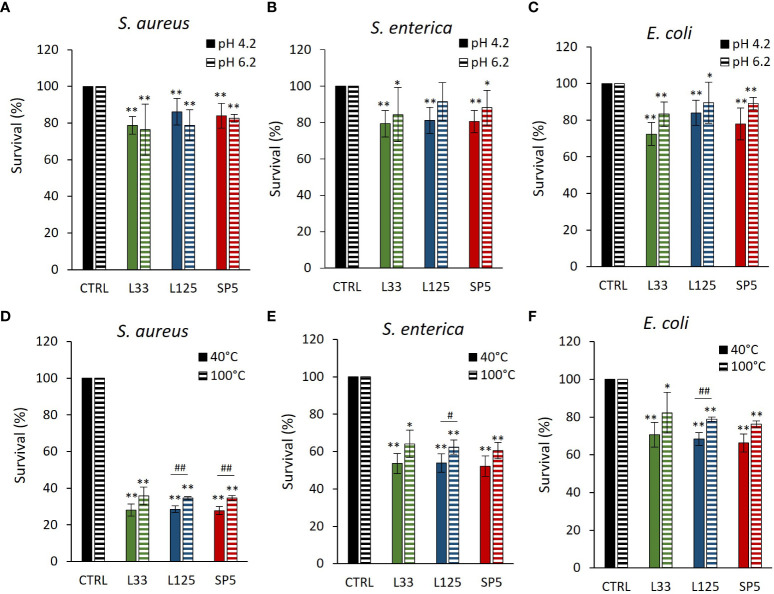
Effect of **(A–C)** pH (4.2, 6.2) and **(D–F)** heat treatments (40, 100°C) on the activity of the CFCS derived from *Lp. plantarum* L125, *Lp. pentosus* L33 and *Lc. paracasei* SP5 against *S. aureus, S. enterica* and *E. coli* after 24 h incubation at 37°C. Viability was determined spectrophotometrically at 620 nm. Data are presented as the mean ± standard deviation of three independent experiments. *p < 0.05; **p < 0.01 compared to control [pathogens treated with heat-treated MRS (40, 100°C) or adjusted at pH 4.2, 6.2]. ^#^ *p* < 0.05; ^##^ *p* < 0.01, compared to non-heat-treated CFCS.

The antimicrobial capacity of metabolites produced by the lactobacilli was tested against planktonic cells of *S. aureus, S. enterica* and *E. coli*. More specifically, pathogens were treated with CFCS at native pH (4.2) or adjusted pH (6.2). As shown in **Figures 3A–C**, native CFCS from all tested lactobacilli, reduced pathogen viability (*p* < 0.01), compared to control (MRS adjusted at pH 4.2). Similarly, adjusted CFCS derived from *Lc. paracasei* SP5 and *Lp. plantarum* L125, retained its antimicrobial activity against all pathogens, while treatments with CFCS derived from *Lp. pentosus* L33, inhibited the proliferation of *S. aureus* and *E. coli*, showing no significant effect against *S. enterica.*

Heat-treated CFCS (pH 4.2) was utilized to determine the stability of the bioactive compounds responsible for the antimicrobial effects at denaturing temperature. It was found that the antimicrobial activity of *Lp. pentosus* L33 heat-treated CFCS was not significantly influenced (**Figures 3D–F**). On the other hand, heat treated CFCS derived from *Lp. plantarum* L125 limited pathogen viability significantly less than non-treated CFCS. Interestingly, heat-treated *Lc. paracasei* SP5 CFCS retained its antimicrobial activity against *S. enterica* and *E. coli*, exhibiting decreased activity against *S. aureus* (*p <* 0.05).

### Antibiofilm capacity of CFCS

**Figure 4:**
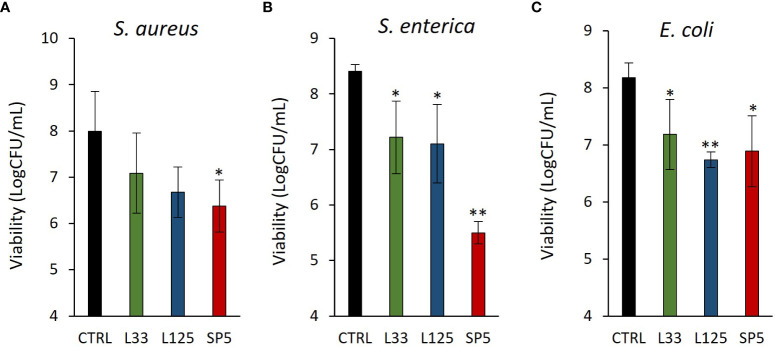
Anti-biofilm activity of CFCS derived from the novel lactobacilli against the enteropathogens **(A)** *S. aureus*, **(B)** *S. enterica* **(C)** and *E*. *coli*. Viability of the biofilm is expressed as Log CFU/mL. Results are expressed as the mean ± standard deviation of three independent experiments. *p < 0.05, **p < 0.01 compared to untreated control.

**Figure 5:**
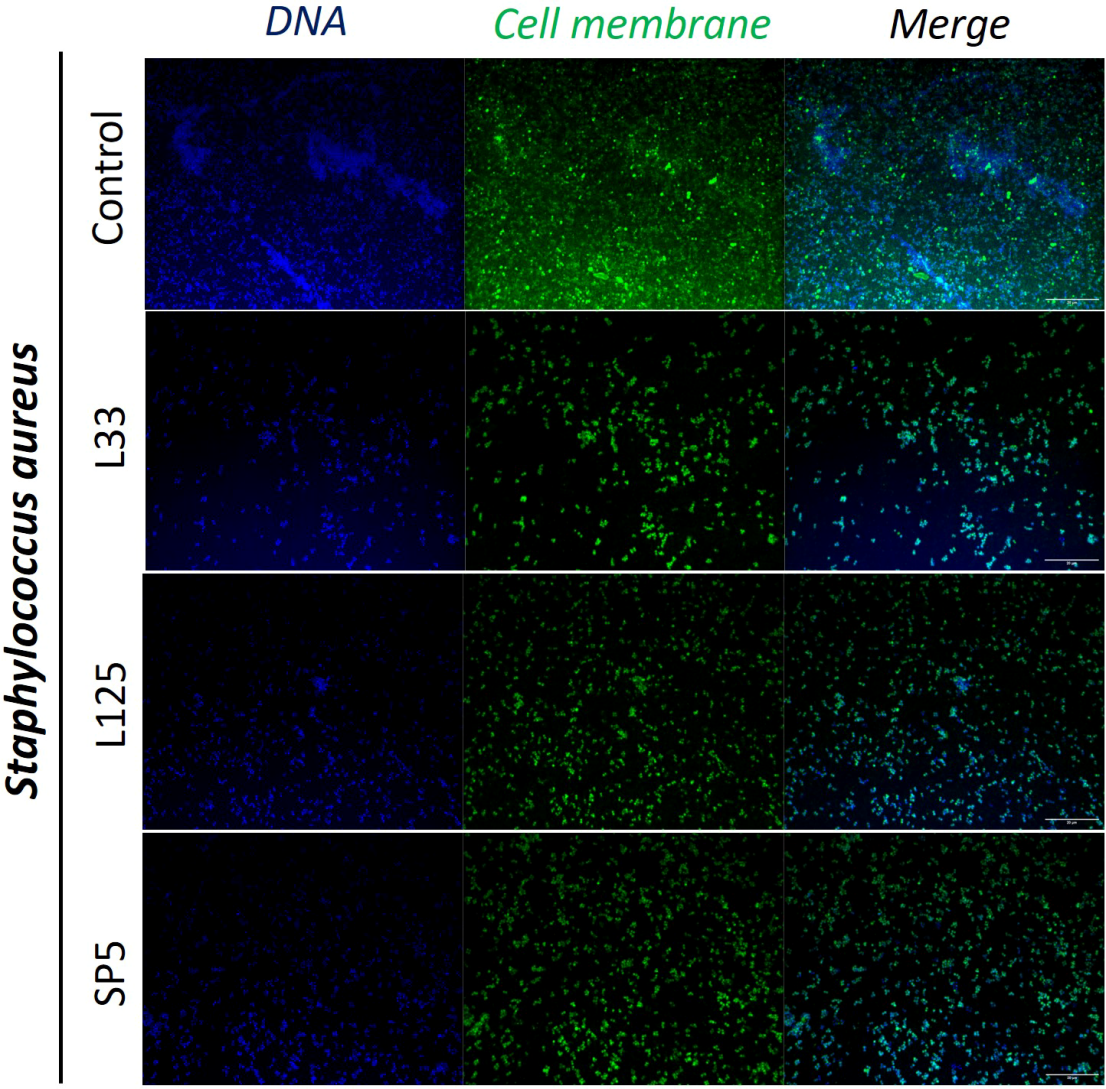
Representative images of *S. aureus* cells organized in biofilms, after 24 h incubation in TSB (control) or adjusted CFCS (50% v/v, pH 6.2) derived from *Lp. pentosus* L33, *Lp. plantarum* L125 or *Lc. paracasei* SP5. Pathogen cell membranes were stained with CFSE (green) and DNA with Hoechst 33342 (blue) (scale bar, 20μm).

**Figure 6:**
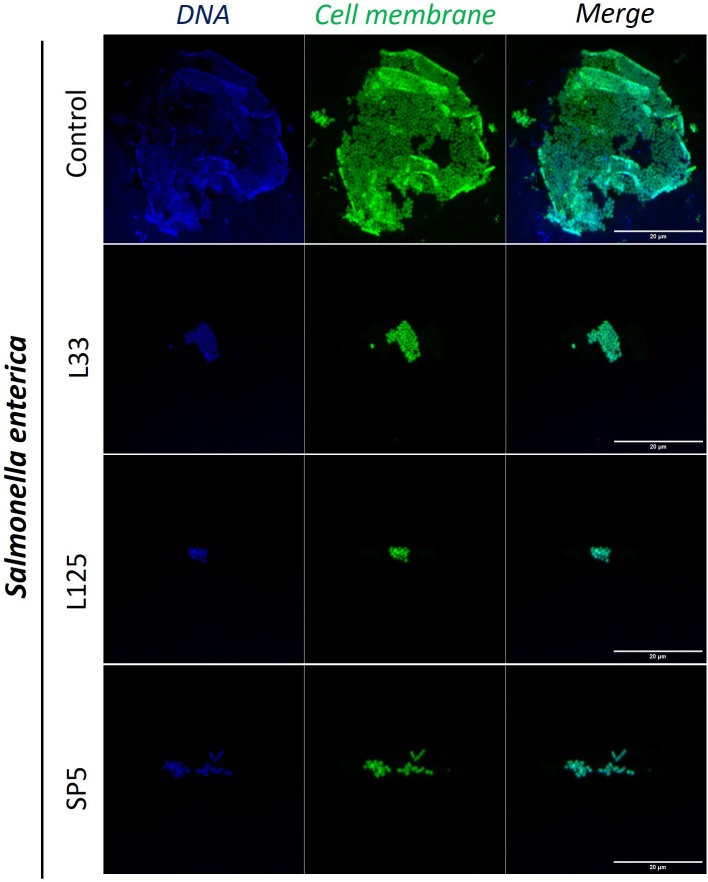
Representative images of *S. enterica* cells organized in biofilms, after 24 h incubation in TSB (control) or adjusted CFCS (50% v/v, pH 6.2) derived from *Lp. pentosus* L33, *Lp. plantarum* L125 or *Lc. paracasei* SP5. Pathogen cell membranes were stained with CFSE (green) and DNA with Hoechst 33342 (blue) (scale bar, 20μm).

**Figure 7:**
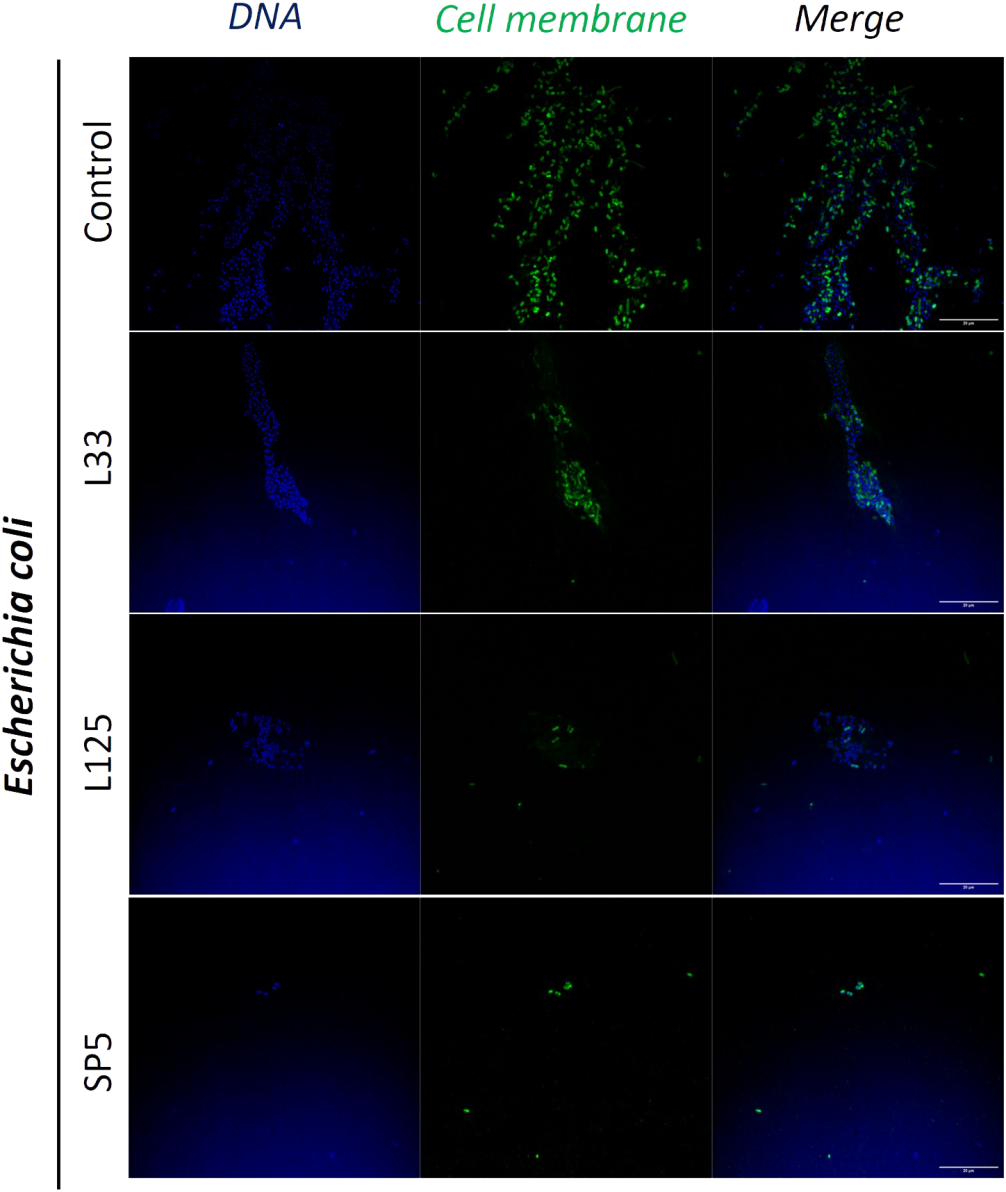
Representative images of *E. coli* cells organized in biofilms, after 24 h incubation in TSB (control) or adjusted CFCS (50% v/v, pH 6.2) derived from *Lp. pentosus* L33, *Lp. plantarum* L125 or *Lc. paracasei* SP5. Pathogen cell membranes were stained with CFSE (green) and DNA with Hoechst 33342 (blue) (scale bar, 20μm).

Next, we determined the ability of CFCS-derived metabolites to inhibit biofilm formation of pathogens after co-incubation for 24 h at static conditions, utilizing a CFU determination assay (**Figure 4**), confocal microscopy (**Figures 5**-**7**), and the crystal violet assay (**Supplementary Figure 2**). *S. aureus* biofilm viability was significantly impaired by *Lc. paracasei* SP5 CFCS treatment (*p* < 0.05, **Figure 4A**), while it was only marginally decreased by *Lp. pentosus* L33 and *Lp. plantarum* L125. On the other hand, biofilm viability of *S. enterica* and *E. coli* was significantly reduced by treatments with CFCS derived from all three lactobacilli (**Figures 4B, C**). The most prominent effect was induced by *Lc. paracasei* SP5 against *S. enterica*, reaching a 3-log reduction (*p* < 0.05) (**Figure 4B**). Confocal microscopy provided visual evidence of the inhibitory activity of the tested CFCS on biofilm formation, as demonstrated in **Figures 5**–**7**. Biofilm biomass was also significantly reduced after treatments with CFCS, derived from the lactobacilli (**Supplementary Figure 2**).

### Measurement of the antibiofilm potential of CFCS *via* investigation of the expression levels of biofilm formation-related genes

**Figure 8:**
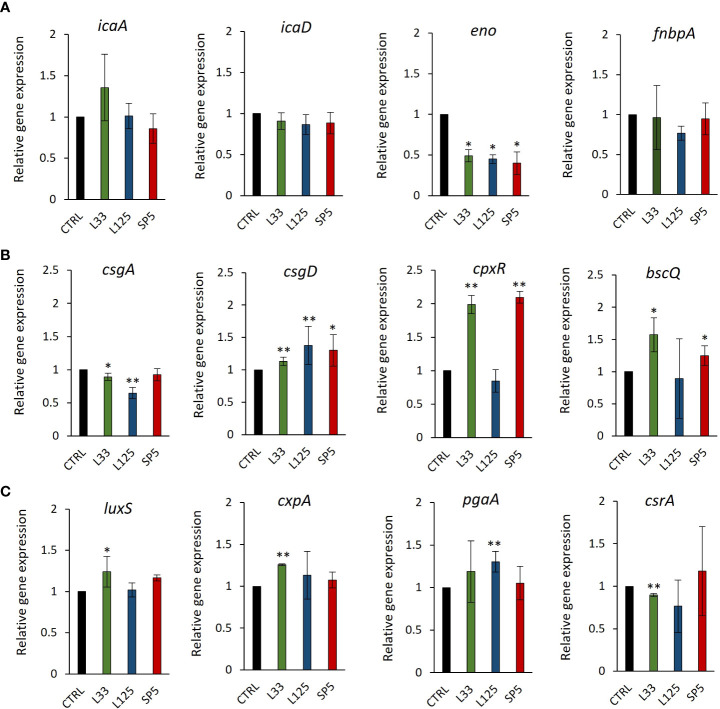
Expression levels of genes involved in biofilm formation after CFSE simulation for 24 hours. **(A)** *S. aureus*, **(B)** *S. enterica* and **(C)** *E*. *coli*, were treated with CFCS derived from *Lp. plantarum* L125, *Lp. pentosus* L33 or *Lc. paracasei* SP5 and TSB at a dilution ratio of 1:2 (or 50% v/v), for 24 h. The control sample was treated with uninoculated MRS medium and TSB (1:2). The data presented are the mean ± standard deviation of three independent experiments. *p < 0.05; **p < 0.01, compared to control.

To delve deeper into the ability of CFCS to inhibit biofilm formation, we determined the possibility of the lactobacilli-derived metabolites to modulate the expression levels of biofilm-related genes at the transcriptome level, after 24 h of co-incubation. The selected genes modulate adhesion of pathogens on hydrophobic surfaces and auto-aggregation, as well as the production and transport of polysaccharides that comprise the protective extracellular capsule of biofilms (**Table 1**). Concerning *S. aureus*, CFCS derived from all three strains significantly lowered the expression of enolase, an adhesion-related moonlighting protein that mediates attachment on inorganic and organic surfaces, inducing no significant effect on the expression levels of the biofilm formation regulator complex *icaA/icaD* or the *FnbpA* adhesin (**Figure 8A**). In the case of *S. enterica*, significant downregulation on the expression of *csgA*, a gene involved in the production of the major curlin subunit, was recorded, after treatments with CFCS derived from *Lp. pentosus* L33 and *Lp. plantarum* L125 (**Figure 8B**). Concomitantly, the same treatments induced a significant upregulation of *csgD* and *cpxR*. Both genes code for transcriptional regulators responsible for adhesion on hydrophobic surfaces and biofilm formation (**Table 1**). Incubation of *E. coli* with CFCS derived from *Lp. pentosus* L33 upregulated the production of *luxS*, involved in quorum sensing, and of the adhesin-coding gene *cpxA* (**Figure 8C**) and downregulated the negative biofilm formation regulator, *csrA. Lp. plantarum* L125 CFCS upregulated *pgaA*, a gene that stimulates the production of the exopolysaccharide biofilm matrix.

### Identification of genes and genetic clusters coding for bacteriocins and antimicrobial metabolites

**Figure 9:**
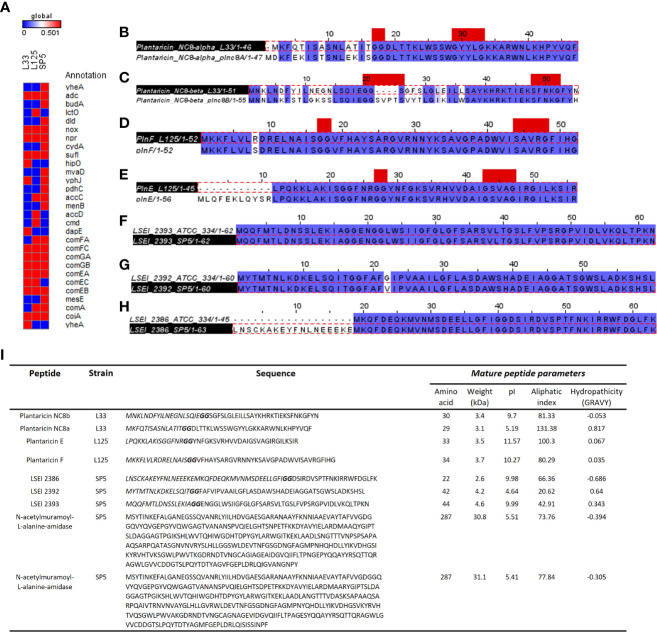
Annotation of genes and gene clusters involved in the antimicrobial capacity of the tested lactobacilli. **(A)** Presence/absence matrix including genes involved in the production of secondary metabolites, lactic acid, ethanol, and hydrogen peroxide presenting antimicrobial activity, as well as genes included in competency clusters (*Com*F, *Com*G, *Com*E). Annotation of WGS was performed in the KEGG database. **(B–H)** Sequence alignment of putative bacteriocin pro-peptides identified in the genome of **(B, C)** *Lp. pentosus* L33, **(D, E)** *Lp. plantarum* L125 and **(F–H)** *Lc. paracasei* SP5 with homologous bacteriocins of closely related strains. The GG cleavage motif and the GxxxG or SxxxG dimerization motifs are denoted in red. The alignments were performed with ClustalW and Jalview was utilized for the visualization of results. **(I)** Table presenting the sequences of putative bacteriocins identified in WGS of strains using BAGEL4 and Blastp+. The GG motif is highlighted in bold, and the N-terminal signal sequence in italics. The physicochemical properties of the putative peptides were determined using ProtParam.

Comparative genomics and annotation algorithms were utilized to expand the bioinformatic analysis on the antimicrobial capacity of the putative probiotic strains. The KEGG database was utilized to investigate the presence of non-proteinaceous compounds that could contribute to pathogen inhibition. More specifically, genes coding for enzymes regulating the production of antimicrobial metabolites, including lactic acid (L-/D- lactate dehydrogenase), ethanol (decarboxylase, alpha-acetolactate decarboxylase, diphosphomevalonate decarboxylase), hydrogen peroxide (NADH oxidase, multicopper oxidase), were identified. Interestingly, competence gene clusters, responsible for nucleic acid uptake after cell disruption were also found conserved in the genome of *Lp. pentosus* L33, *Lp. plantarum* L125 and *Lc. paracasei* SP5 (**Figure 9A**).

Furthermore, we sought to detect genes coding for antimicrobial proteins and/or peptides in the genome of the strains. To this aim, analysis with BAGEL4 revealed clusters containing putative bacteriocins (**Supplementary Figure 3**). To further determine the functionality of peptides and their accessory proteins, comparative analysis with characterized peptides, ensued. More specifically, *Lp. pentosus* L33 carries two loci, whose structure resembles that of bacteriocin clusters (**Supplementary Figure 3A**). One of these clusters includes genes for Plantaricin NC8 chains alpha and beta (core peptides) and Plantaricin A, the pheromone regulating the expression of the cluster. Downstream to these loci, a bacteriocin immunity protein (orf00024) and genes coding for proteins responsible for GG-leader motif cleavage and transport (orf00047, orf00048, orf00053), were identified. Sequence analysis of the core peptides revealed that chain alpha carries a SxxxG motif and chain beta a GxxxG motif possibly involved in the dimerization of the two partners. To predict the functionality of the peptides, Plnc8a and Plnc8b were aligned against the functionally characterized sequences of strain *Lp. plantarum* NC8 (Maldonado et al., 2003). The mature peptides (cleaved at the GG motif) of Plnc8a share 100% sequence and structural conservation. Similarly, Plnc8b mature peptides share a 79.31% sequence identity and structural conservation (**Figures 9B, C**). Of note, Plnc8b encoded by *Lp. pentosus* L33 carries only one dimerization motif, while its homologous sequence contains two. Thus, dimer formation for the active form of the peptide could be impaired. Concerning the physicochemical properties of the peptides, Plnc8a weighs 3.5 kDa and Plnc8b 3.4 kDa with a theoretical pI of 9.87 and 9.7, respectively (**Figure 9I**).

A cluster coding for Plantaricin EF was identified in the genome of *Lp. plantarum* L125 (**Supplementary Figure 3B**). This locus also contains a gene to produce the pheromone Plantaricin K. Furthermore, it contains an immunity protein (plnL; orf00025) and other transport/immunity proteins (orf00002, HlyD, orf00016). Sequence analysis of the core peptides revealed the presence of the dimerization motif GxxxG in both mature peptides. To predict their functionality, sequences derived from Bactibase of *Lp. plantarum* C11 (Diep et al., 1996), were used. It was found, that the mature PlnE and PlnF peptides coded by *Lp*. *plantarum* L125, present 100% sequence and structural identity with the respective peptides derived from *Lp*. *plantarum* C11 (**Figures 9D, E**). PlnE is a 3.5 kDa protein, with a theoretical pI of 11.57, and PlnF weighs 3.7 kDa, with a theoretical pI of 10.27 (**Figure 9I**).

**Figure 10:**
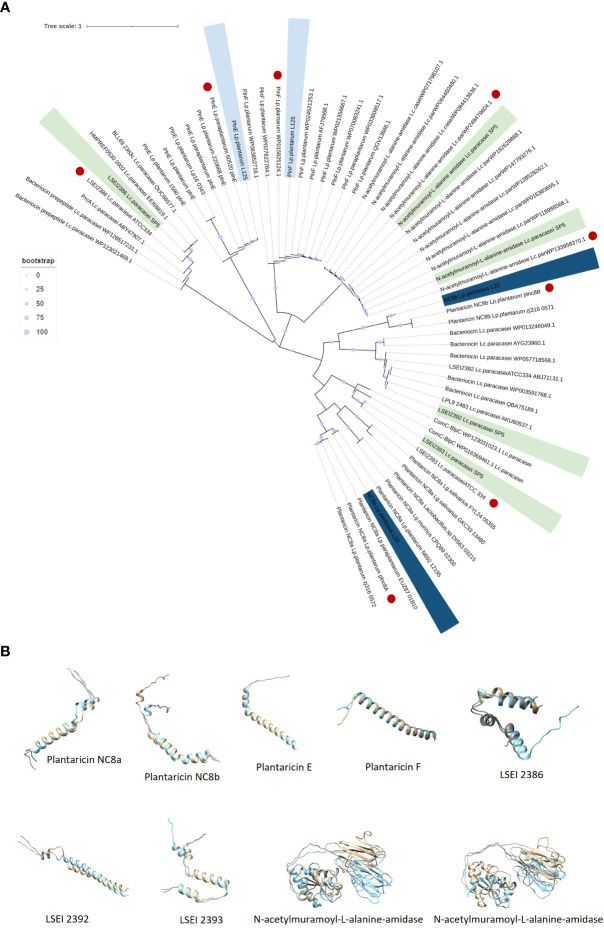
**(A)** Unrooted Neighbor-Joining tree containing putative bacteriocin sequences (pro-peptides) located in the whole genome sequence of the five lactobacilli and homologous sequences of closely related LAB. Sequences with the highest sequence identity and structure conservation with the putative bacteriocins are denoted in red. **(B)** Structure superimposition of putative bacteriocins derived from the novel lactobacilli (blue) against functionally characterized peptides derived from the literature (light yellow). Structural predictions were made using ColabFold and structural superimpositions with Chimera 1.16.

*Lc. paracasei* SP5 harbors multiple core peptides with putative antimicrobial activity (**Supplementary Figure 3C**). More specifically, the putative bacteriocin LSEI 2386 was annotated using BAGEL4 alongside putative transport and immunity proteins LanT, and Enterocin A immunity proteins. LSEI 2386 (2.5 kDa, pI 8.34) presents 100% sequence and structural conservation to the respective peptide annotated in the genome of *Lc. casei* ATCC 334 (Kuo et al., 2013), now belonging to the *Lc. paracasei* species (Zheng et al., 2015). To determine the presence of proteins specific for the modification and export of the LSEI 2386 peptide, sequences derived from the same strain were queried against the WGS of *Lc. paracasei* SP5. Genes coding for an ABC transporter (LSEI 2384) and an accessory secretion protein (LSEI 2381) were annotated upstream of the core peptide with 99% sequence identity (**Figures 9F–H**). Furthermore, the putative bacteriocin peptides LSEI 2392 (4.28 kDa, pI 4.64) and LSEI 2393 (4.6 kDa, pI 9.99), were also identified in the genome of the strain, presenting 98% and 100% sequence identity with the characterized peptides coded by *Lc. casei* ATCC334 (Kuo et al., 2013). The accessory secretion protein LSEI 2389 was also annotated upstream of the genes coding for the core peptides, with 100% identity with the sequence derived from *Lc. casei* ATCC334. Lastly, two genes coding for N-acetylmuramoyl-L-alanine-amidase, an enzyme responsible for peptidoglycan degradation were pinpointed in the genome of *Lp. paracasei* SP5. Although BAGEL4 annotated these loci as Enterolysin A, further analysis revealed greater similarity with the enzyme N-acetylmuramoyl-L-alanine-amidase coded by closely related lactobacilli (> 90%). Furthermore, domain analysis with InterPro showed that both annotated sequences carry an amidase domain, including a catalytic site, a substrate binding site and a Zinc binding site, characteristic of this enzyme family. Both proteins weigh around 31 kDa, possessing a theoretical pI of 5.41 (**Figure 9I**). The putative bacteriocin peptides annotated in the genome of the novel strains, and homologous proteins from other lactobacilli were aligned and utilized for the construction of an unrooted tree, using the neighbor joining method and 1000 bootstrap replications (**Figure 10A**). Furthermore, the structure of the putative bacteriocin pro-peptides was predicted using AlphaFold2, and their tertiary structure was superimposed to that of homologous proteins, whose function was experimentally validated (**Figure 10B**).

Finally, the genome sequences of the three LAB strains were scoured for the presence of genes involved in quorum quenching. The Uniprot database was searched for annotated sequences coding for N-acyl homoserine (AHL) lactonase, AHL acylases and AHL oxidoreductases. Among these enzymes, only AHL lactonases were characterized in lactobacilli. Thus, two sequences (A0A7Z2PEZ0 and A0A0M3QBV3) identified in *Lp. plantarum* were queried against the genome of the strains. It was found that *Lp. plantarum* L125 possesses one locus presenting sequence identity of 100% and 98% with the queries, while *Lp. pentosus* L33 also codes for a homologous gene, that however, presents lower percentage identity (77% and 78%) (**Supplementary Table 1**).

## Discusssion

Probiotics are described as microorganisms that can be beneficial to the consumer when administered in adequate amounts (Hill et al., 2014). The health-promoting properties of microorganisms are thought to be strain specific. Indeed, members of the same species can elicit either pro-inflammatory or anti-inflammatory activity, modulate the gut microbiota differentially, or induce targeted antimicrobial effects (Ramos et al., 2013; Su et al., 2017). In this context, we determined the inhibitory activity of three putative probiotics against *Staphylococcus aureus, Salmonella enterica* ssp *enterica* serovar Enteritidis and *Escherichia coli*, three common enteropathogens that are responsible for serious intestinal or extraintestinal complications, following well-established *in silico* and *in vitro* protocols.

First, we determined the ability of lactobacilli cultures to limit pathogen viability using the agar well diffusion assay. All tested lactobacilli produced clear zones in the swabbed agar, with more profound effects being recorded against *S. aureus* and *E. coli* (**Figure 1**). This method is a common approach to assess the susceptibility of bacteria to viable cultures, crude extracts, or isolated compounds (Balouiri et al., 2016). Indeed, previous studies have utilized this assay to elucidate the inhibitory activity of novel lactobacilli isolated from fermented foodstuffs against foodborne pathogens (Prabhurajeshwar and Chandrakanth, 2017). Next, we sought to evaluate the antimicrobial activity of viable lactobacilli against planktonic pathogenic cells in co-culture, showing that *S. aureus* and *E. coli* were susceptible to the putative probiotics. Interestingly, phenomena of reciprocal inhibition were recorded in co-incubation of *S. enterica* with *Lp. pentosus* L33 and *Lc. paracasei* SP5, as *S. enterica* significantly limited their viability *in vitro* (data not shown). This model is used to investigate phenomena of nutrient antagonism and the contribution of inducible bacteriocins in the antimicrobial activity of bacterial strains (Chanos and Mygind, 2016). The production of bacteriocins and bacteriocin-like peptides can be auto-induced or triggered by the presence of target microorganisms, subsequently acting as quorum sensing or antimicrobial peptides in suspension (Arqués et al., 2015). In this context, plantaricins derived from *Lp. plantarum* strains (Rojo-Bezares et al., 2007; Wu et al., 2021) and gasserin A produced by *Lactobacillus gasseri* EV1461 (Maldonado-Barragán et al., 2016) were detected in greater amounts in co-culture with target microorganisms. The ability of strains to produce inducible bacteriocins is especially important during the *in-situ* fermentation process, as they can alter matrix microbiota composition (Settanni et al., 2005), therefore influencing the organoleptic properties of foods, as well as their resilience to contamination (Devi et al., 2014).

Host colonization is a decisive step during early infection stages, mediated by cell surface molecules, and thus its prevention presents clear advantages (Tuo et al., 2018). Similar to pathogens, the cell surface of lactobacilli is decorated with proteins and polysaccharides that can mediate adhesion onto abiotic and biotic surfaces, including host mucosa (Salas-Jara et al., 2016). Therefore, lactobacilli could compete with pathogens for finite binding sites on host mucosa and epithelia. In this work, we determined the ability of the tested lactobacilli to co-aggregate and exclude pathogens from HT-29 monolayers. Among the tested strains, *Lc. paracasei* SP5 effectively co-aggregated and managed to significantly limit adherence of the pathogenic cells. The adhesion capacity of this putative probiotic strain has been previously reported, while proteins involved in attachment on host mucosa have been predicted in its genomic sequence (Kiousi et al., 2022). Competitive exclusion may be induced by non-specific interactions with the epithelium and pathogens or by competition for host receptors by homologous proteins expressed at the surface of lactobacilli and pathogenic species that determine their adhesion in the gut niche, as reported in previous studies (Chen et al., 2007; van Zyl et al., 2020). Additionally, non-protein compounds can also participate in this phenomenon, as evidenced in a study where elimination of surface-bound proteins did not affect the ability of *Lp. plantarum* 423 to exclude *Clostridium sporogenes* LMG 13570 or *Enterococcus faecalis* LMG 13566, from Caco-2 monolayers (Ramiah et al., 2008). Taking these findings together, we hypothesize that the exclusion of pathogens recorded mainly for *Lc. paracasei* SP5, may be attributed to both competition for binding sites on eukaryotic cells and interference by co-aggregation. A more detailed investigation of the competitive exclusion capacity of *Lc. paracasei* SP5 will be performed in the future.

CFCS derived from lactobacilli strains is a rich source of bactericidal and bacteriostatic metabolites, including lactic acid and other acidic compounds, as well as bacteriocin and bacteriocin-like peptides, exopolysaccharides, and other small molecules, such as ethanol and hydrogen peroxide (Prabhurajeshwar and Chandrakanth, 2017). LAB produce high amounts of lactic acid, and thus matrix acidification majorly contributes to the antimicrobial capacity of lactobacilli CFCS, as most human pathogens cannot withstand extremely low pH values. To test the role of matrix acidification in the recorded antimicrobial phenotype, we examined the ability of CFCS to limit viability of the enteropathogens at pH values of 4.2 and 6.2. Interestingly, treatments with CFCS (pH 4.2) significantly limited viability of pathogens compared to the negative control (MRS adjusted at pH 4.2). To understand this finding, we need to examine how the acidic pH influences cell survival. It has been reported that exposure to low pH disrupts membrane integrity, thus, the heightened antimicrobial activity at this condition could be explained by the synergistic action of low pH with other factors acting with the same mechanism, including bacteriocins (Mason et al., 2006). Then, we sought to determine whether the CFCS retained its antimicrobial activity at pH 6.2. The antimicrobial activity was, indeed, sustained against *S. aureus* and *E. coli* planktonic cells treated with CFCS derived from the lactobacilli. However, the viability of *S. enterica* was not significantly decreased after treatments with adjusted CFCS (pH 6.2), derived from *Lp. pentosus* L33 or *Lp. plantarum* L125. Lastly, we used heat treatments to determine the stability of the bioactive compound(s) at denaturing conditions. Among the tested strains, the inhibitory capacity of the heat-treated CFCS derived from *Lp. plantarum* L125 was completely abolished, indicating the presence of a bactericidal heat-labile molecules, including protein(s). On the other hand, *Lp. pentosus* L33 was still able to exert antimicrobial activity against all three pathogens, possibly alluding to the fact that the recorded antimicrobial activity of *Lp. pentosus* L33 CFCS may not be attributed to an antimicrobial protein, but rather depend on the presence of small compounds or of other non-protein molecules. These results do not exclude the possibility of the strain to code for inducible bacteriocins, only in the presence of target microorganisms. Curiously, heat-treated *Lc. paracasei* SP5 CFCS, retained its antimicrobial activity against *S. enterica* and *E. coli.* This pathogen-specific effect could be attributed to the activity of heat-resistant bacteriocins. Indeed, bacteriocins sustaining their antimicrobial potential at 121°C have been described before, for *Lp. paracasei* ssp*. paracasei* LP5, *L. brevis* LP9 (Iseppi et al., 2019) and *Lc. casei* ATCC334 (Kuo et al., 2013).

Pathogen biofilm formation is a mechanism that supports resilience in hostile environments. Biofilms are complex three-dimensional structures formed on biotic or abiotic surfaces, that are comprised by bacteria enclosed in a polysaccharide shell, often including extracellular DNA. Bacteria that comprise these communities present lower metabolic and replication rates (Yin et al., 2020). The polysaccharidic extracellular matrix, the compactness of the structure and the limited metabolic activity of the strains, increase resistance to common antibiotics (Yin et al., 2020). In this work, we examined the antibiofilm potential of CFCS derived from lactobacilli cultures at the late exponential phase. It was shown that *S. enterica* and *E. coli* biofilm viability was influenced by all treatments, while *S. aureus* biofilm viability was only significantly limited by *Lc. paracasei* SP5. Similarly, antibiofilm activity of CFCS derived from probiotic LAB was also reported in previous studies against *S. aureus* (Lee et al., 2020), uropathogenic *E. coli* U12 (Mekky et al., 2022) and *S. enterica* subsp. *enterica* serovar Enteritidis (Tazehabadi et al., 2021). Furthermore, confocal microscopy was utilized to visually examine biofilm mass and morphology after co-incubation of the pathogens with CFCS. Of note, the antibiofilm activity of CFCS was abolished at a dilution ratio of 1:5 or at a concentration of 20% v/v (data not shown). Confocal microscopy imaging can be also used to detect the extracellular matrix of biofilms, including extracellular DNA (eDNA), while different dyes are used to distinguish live/dead cells (Reichhardt and Parsek, 2019). Here, staining with CFSE was utilized to detect live cells, while DNA staining with Hoechst was performed to determine residual nucleic acids after cell lysis and the presence of eDNA. Consistent to this, confocal microscopy was employed for the investigation of the antibiofilm capacity of probiotic streptococci against pathogens of the upper respiratory tract, showing that treatments limited biofilm mass and viability (Bidossi et al., 2018).

To delve deeper into the ability of CFCS to interfere with biofilm formation, we examined changes in the expression levels of genes involved in processes including aggregation, surface attachment and the production of exopolysaccharide capsule. CFCS derived from all lactobacilli significantly downregulated the production of enolase, a protein involved in the adhesive phenotype. A previous proteomic study has shown that *S. aureus* recycles cytoplasmic proteins with moonlighting functions, including enolase, to produce the extracellular matrix, rather than employing a dedicated biofilm protein (Foulston et al., 2014). In this context, norgestimate, an acetylated progestin, limited *S. aureus* biofilm formation by inducing changes at the transcriptome and proteome level, including enolase downregulation (Yoshii et al., 2017). Concerning expression changes to *S. enterica, Lp. pentosus* L33 and *Lp. plantarum* L125 significantly downregulated *csgA*, a gene involved in fibriae production and upregulated *csgD* a transcriptional regulator of biofilm formation. These genes are conserved in *E. coli* and *S. enterica*, being encoded in two different operons that are regulated by several transcriptional factors. CsgD upregulates the production of fibriae by inducing the *csgBA* operon, however the expression of both *csgBA* and *csgDEFG* is regulated by CpxR (Prigent-Combaret et al., 2001; Sokaribo et al., 2020). Treatments with CFCS induced the expression of these genes, possibly alluding to an adaptation mechanism to ensure survival of the remaining cells after the bactericidal activity of metabolites in the CFCS. Similarly, *Lp. pentosus* L33 promoted the transcription of *luxS*, which is involved in quorum sensing, and of *cpxA*, a hydrophobic surface adhesin. Furthermore, *Lp. pentosus* L33 decreased the production of a negative regulator of biofilm formation, *csrA.* Taken together, these findings highlight that the antimicrobial potential of CFCS majorly contributes to the antibiofilm capacity recorded, possibly supported by changes in the expression of key genes regulating biofilm formation in a strain- and pathogen-specific manner. In agreement to this, a previous study investigated the effects of CFCS derived from *L. kefiranofaciens* DD2 onto the viability and biofilm formation of oral pathogens, showed that the supernatants exhibited both bactericidal activity and capacity to modulate biofilm formation by modifying expression of biofilm-related genes (Jeong et al., 2018).

The genetic basis of the antimicrobial and antibiofilm phenotype of *Lp. pentosus* L33, *Lp. plantarum* L125 and *Lc. paracasei* SP5 was investigated using a series of interconnected approaches. The WGS of the strains was scoured for loci coding for putative bacteriocin peptides, or quorum quenching signals. Additionally, pathway analyses were utilized to detect enzymes and metabolic clusters responsible for the production of secondary metabolites involved in the inhibitory activity of strains. *Lp. pentosus* L33 possesses a plantaricin NC8 cluster, that presents high identity to the characterized cluster possessed by *Lp. plantarum* NC8 (Maldonado et al., 2003). Plantaricin NC8 is a potent, inducible class IIb bacteriocin that can diffuse through the peptidoglycan wall, causing cell membrane permeabilization. This dipeptide was previously shown to possess antimicrobial activity against *Staphylococcus* spp (Bengtsson et al., 2020) and *Salmonella* spp (Jiang et al., 2016). *Lp. plantarum* L125 harbors a full cluster coding for Plantaricin EF, a class IIb, two-peptide (PlnE and PlnF), heat-stable bacteriocin. These two peptides adopt an antiparallel conformation in space, allowing for the formation of helix-helix interactions between the SxxxG motif of PlnE and the GxxxG motif of PlnF (Ekblad et al., 2016). The dimers are then embedded in the membrane of target pathogens, causing the formation of pores and the subsequent dissipation of transmembrane electrical potential (Moll et al., 1999). The mature peptides coded by *Lp. plantarum* L125 present 100% sequence identity and high structural conservation with homologous peptides coded by *Lp. plantarum* C11, whose antimicrobial activity has already been described (Diep et al., 1996). Interestingly, a homologous gene coding for an AHL lactonase was also identified. This enzyme is responsible for the catabolism of the lactone ring of AHL, a quorum sensing signal used by biofilm producers, including *S. aureus* and some *E. coli* strains, but not *S. enterica*, therefore resulting in quorum quenching and the disruption of biofilm formation (Prazdnova et al., 2022). Quorum quenching is not a common mechanism for pathogen control among LAB; however, studies have showed that culture supernatants derived from *Lc. rhamnosus, Limosilactobacillus fermentum* or *Lactococcus lactis* possess AHL-degrading ability (Prazdnova et al., 2022). *Lc. paracasei* SP5 contains clusters coding for several putative bacteriocins. More specifically, it contains full clusters for the heat stable peptides LSEI 2386, LSEI 2392 and LSEI 2393, that present high sequence identity and structural conservation with sequences derived from *Lc. paracasei* ATCC 334. LSEI 2386 was assigned as a putative pheromone, responsible for the induction of bacteriocin-producing clusters, exhibiting limited antimicrobial activity against *Listeria spp* (Kuo et al., 2013). Peptide LSEI 2393 presents sequence motifs, structural and physicochemical characteristics of bacteriocins, however no antimicrobial activity against *Listeria* spp or closely related lactobacilli was recorded (Kuo et al., 2013). Of note, these peptides are extremely heat tolerant, resisting denaturation at temperatures as high as 121°C. This finding could provide an explanation for the selective antimicrobial activity of the strain against *S. enterica* and *E. coli* after heat treatments, however further experimental validation is required. Additionally, two genes coding for N-acetylmuramoyl-L-alanine-amidase were annotated in the genome of *Lc. paracasei* SP5. This enzyme is involved in peptidoglycan degradation during cell wall recycling; however, studies suggest its broad-spectrum antimicrobial activity (Lopez-Arvizu et al., 2021).

Taking these findings into consideration, the lactobacilli included in this study presented strain- and pathogen-specific activity. Indeed, the emended *Lactobacillus* genus presents high antimicrobial capacity that is attributed to the strain-specific capacity of strains to code for a plethora of bacteriocins or other antimicrobial non-proteinaceous molecules (Collins et al., 2017). Among the three LAB strains, *Lc. paracasei* SP5 presents broad antimicrobial potential, as it was effective in limiting adhesion of all tested pathogens onto HT-29 monolayers, while exhibiting significant co-aggregation, antimicrobial and antibiofilm capacity, with the most profound effect recorded against *S. enterica* biofilm viability (~3 log reduction). Of note, *Lc. paracasei* SP5 presents biotechnological interest, as it has been previously incorporated in the production of fermented chokeberry juice, white brined cheese, and sourdough bread, with elevated organoleptic characteristics (Bontsidis et al., 2021; Plessas et al., 2021; Kazakos et al., 2022). Interestingly, white brined cheese fermented by this strain, exhibited resistance to the growth of coliforms, yeasts and fungi, suggesting the antimicrobial capacity of the strain, *in situ* (Plessas et al., 2021). Future studies will focus on the characterization of the surface proteome and secreted metabolome of *Lc. paracasei* SP5 to elucidate the molecular and cellular mechanisms involved in the antimicrobial activity recorded *in vitro* and *in situ.*

## Conclusions

In this work, the antimicrobial and antibiofilm activity of three potentially probiotic LAB strains derived from traditional, fermented products, *Lp. pentosus* L33, *Lp. plantarum* L125 and *Lc. paracasei* SP5, was evaluated using *in silico* and *in vitro* analyses, against three common human pathogens, *S. aureus, S. enterica* and *E. coli*. Through a series of interconnected approaches, we found that they exerted strain- and pathogen-specific activity. Among the tested strains *Lc. paracasei* SP5 presented the greatest potential against planktonic cells and of biofilms produced by *S. enterica*, a foodborne pathogen, responsible for gastrointestinal infections with serious health complications. Accordingly, it was successful in the competitive exclusion of all enteropathogens from HT-29 monolayers. Interestingly, the genome of the strain carries a repertoire of bacteriocins and other small molecules with putative antimicrobial activity, thus future studies will aim at elucidating the bactericidal activity utilizing multi-omic approaches for the structural and functional characterization of molecules involved in the recorded phenotypes.

## Data availability statement

The datasets presented in this study can be found in online repositories. The names of the repository/repositories and accession number(s) can be found below: https://www.ncbi.nlm.nih.gov/genbank/, JAHKRU000000000.1; https://www.ncbi.nlm.nih.gov/genbank/, JAIGOE000000000.1; https://www.ncbi.nlm.nih.gov/genbank/, JAKJPP000000000.1.

## Ethics statement

This study was approved (24-10/12/2022) by the Institution Review Board of the General University Hospital of Alexandroupolis, Greece.

## Author contributions

MP, MK, and AG designed the study. DK, CE, and VT carried out the experiments. DK, CE, VT, MK, MP, and AG analyzed the data. DK, CE, VT, and AG participated in the writing of the manuscript. SP, MP, MK, and AG contributed to editing and critical reviewing of the manuscript. SP, MP, MK, and AG took charge of the resources. All authors contributed to the article and approved the submitted version.
