# Supplementary figures and images for: Genetic and phenotypic assessment of the antimicrobial activity of three potential probiotic lactobacilli against human enteropathogenic bacteria
Source: Front Cell Infect Microbiol. 2023 Feb 8;13:1127256. doi: 10.3389/fcimb.2023.1127256 (PMC9944596; doi:10.3389/fcimb.2023.1127256)

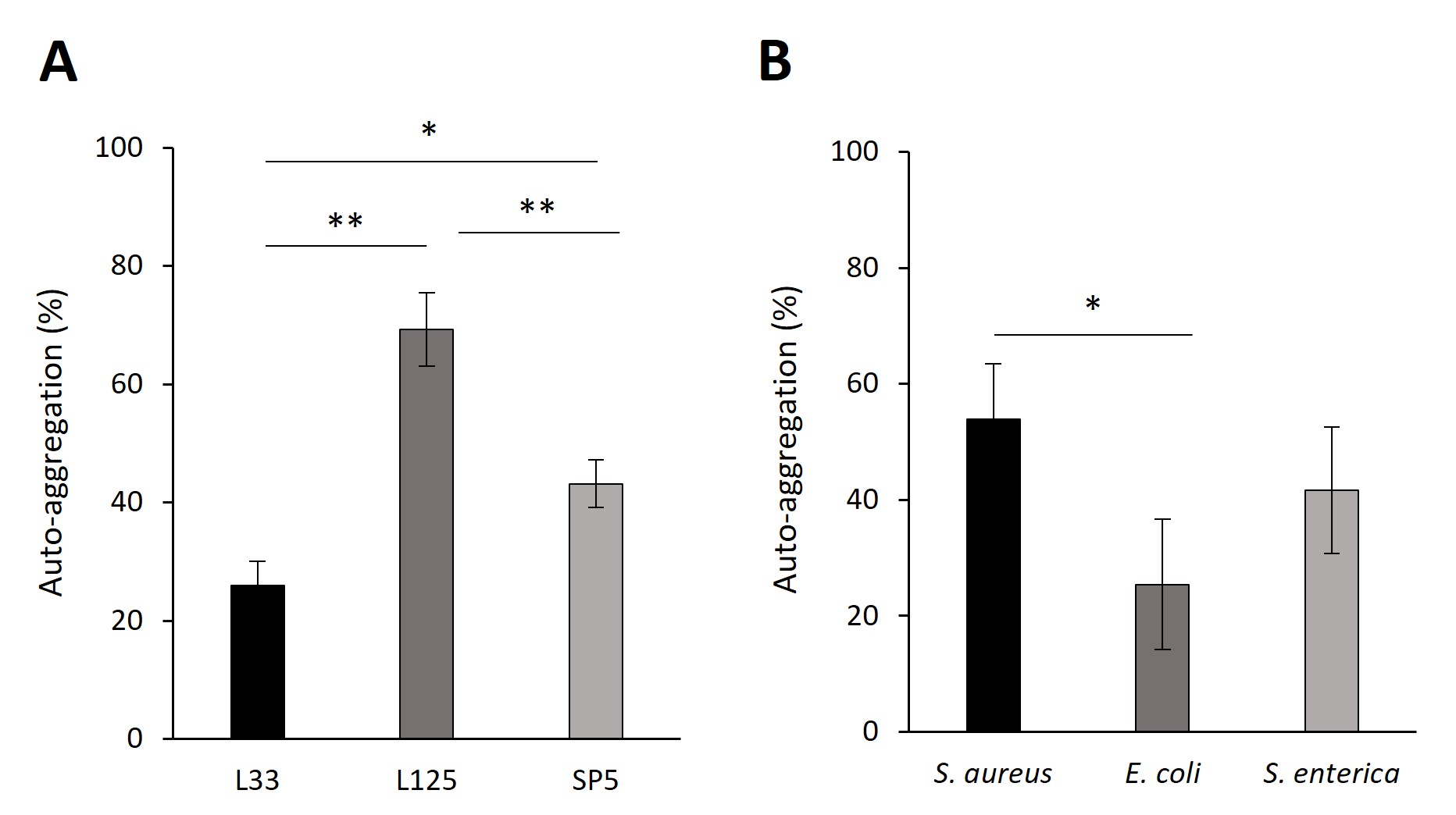

Supplement: Supplementary file 1 [file Image_1.jpeg]

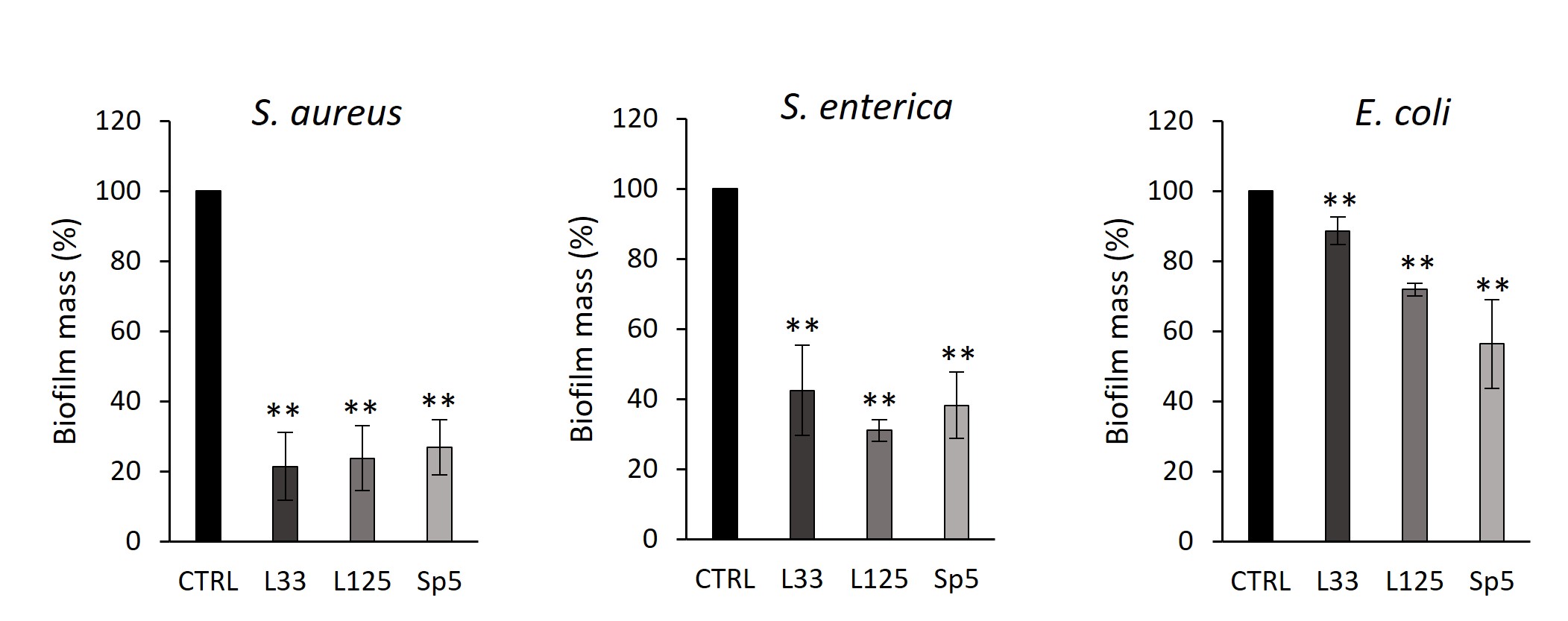

Supplement: Supplementary file 2 [file Image_2.jpeg]

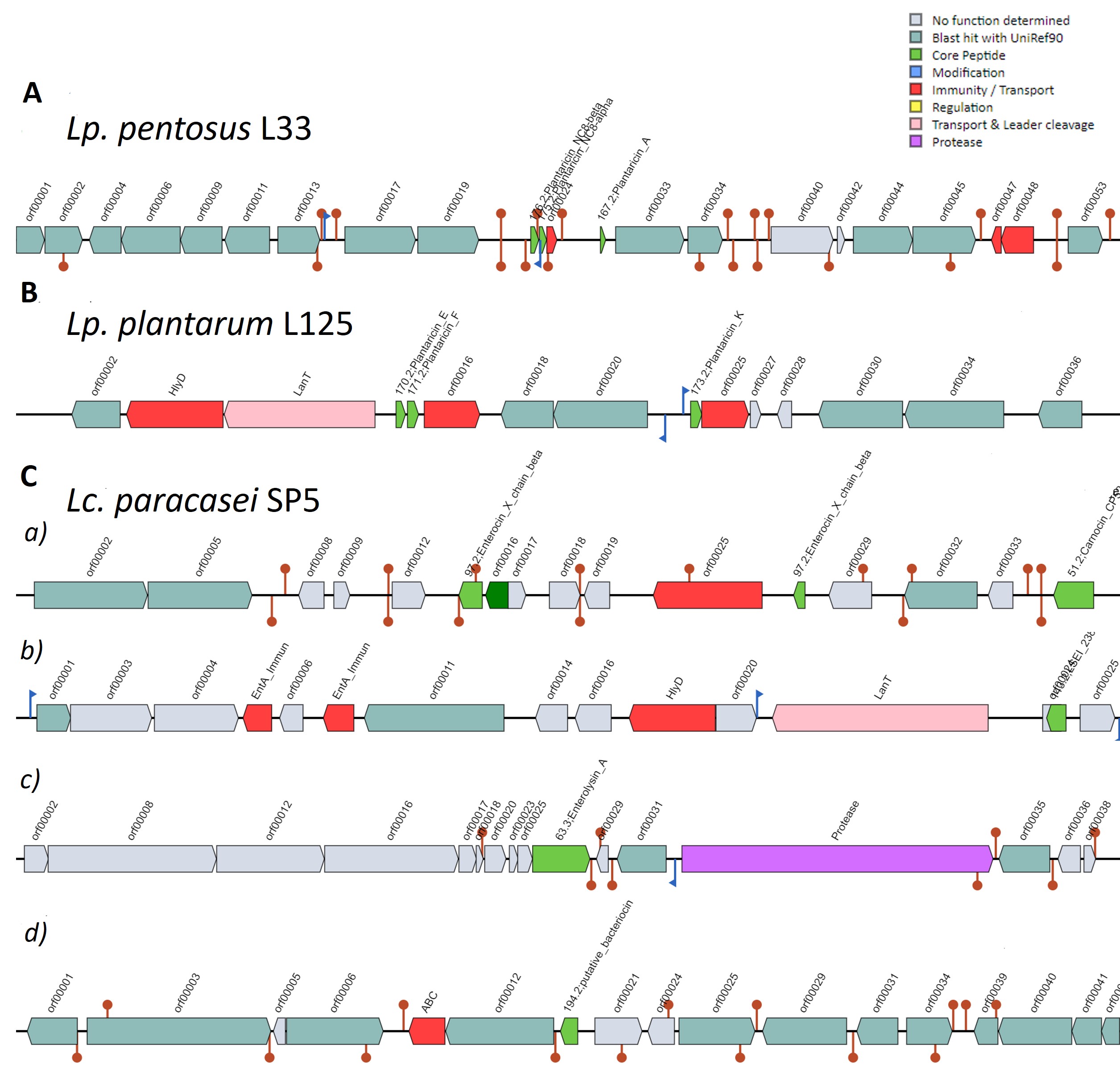

Supplement: Supplementary file 3 [file Image_3.jpeg]
